# Systematic literature review and meta-analysis of lotilaner 0.25% and tea tree oil for *Demodex* blepharitis

**DOI:** 10.3389/fopht.2026.1978403

**Published:** 2026-10-14

**Authors:** Ian Benjamin Gaddie, Mayank A. Nanavaty, Timothy Page, Darrell White, Selina McGee, Pamela E. Theriot, Noemi Fluetsch Brennan, Lisa Bloudek, James Mun, Elizabeth Yeu, H. Burkhard Dick

**Affiliations:** 1 Gaddie Eye Centers, Louisville, KY, United States; 2 Sussex Eye Hospital, University Hospitals Sussex NHS Foundation Trust, Brighton, United Kingdom; 3 Oakland University William Beaumont School of Medicine, Royal Oak, MI, United States; 4 Oakland Ophthalmic Surgery, Birmingham, MI, United States; 5 SkyVision Centers, Westlake, OH, United States; 6 BeSpoke Vision, Edmond, OK, United States; 7 Williamson Azar Vision, Lafayette, LA, United States; 8 Curta Inc, Seattle, WA, United States; 9 Tarsus Pharmaceuticals Inc., Irvine, CA, United States; 10 University Eye Hospital, Ruhr University, Knappschaft Kliniken, Bochum, Germany

**Keywords:** collarettes, *Demodex* blepharitis, *Demodex* mites, erythema, lotilaner, tea tree oil

## Abstract

**Introduction:**

*Demodex* blepharitis (DB) is a common, often underdiagnosed inflammatory eyelid disease caused by *Demodex* mite infestation. Currently, the only FDA-approved treatment for DB is lotilaner ophthalmic solution, 0.25%. Off-label therapies, including tea tree oil or terpinen-4-ol (collectively referred to as TTO) products, have also been used; however, supporting evidence is inconsistent. This systematic literature review and meta-analysis evaluated the treatment effects and quality of evidence for lotilaner ophthalmic solution, 0.25% and TTO products in patients with DB.

**Methods:**

MEDLINE, EMBASE, Cochrane CENTRAL, ClinicalTrials.gov, and ICTRP were searched from inception through July 10, 2025, to identify randomized controlled trials of lotilaner 0.25% or TTO.

**Results:**

Nine randomized controlled trials were included, with four evaluating lotilaner 0.25% versus vehicle and five evaluating TTO versus sham, comprising a total of 1,513 patients. Compared with vehicle, lotilaner 0.25% produced significantly lower mite density (mean difference -1.29 mites/lash at both 2–4 and 6–8 weeks; p<0.0001) and significantly higher odds of mite eradication (OR 4.70 at 2–4 weeks; OR 9.60 at 6–8 weeks), collarette reduction to 0–2 collarettes (OR 8.39 and 9.67), and erythema cure at 6–8 weeks (OR 3.91; all p<0.0001). TTO did not demonstrate a statistically significant difference versus sham for mite density or mite eradication at either timepoint.

**Discussion:**

These findings demonstrate a consistent, statistically significant benefit of lotilaner 0.25% across assessed outcomes compared with vehicle, supporting lotilaner 0.25% as an effective pharmacological treatment for DB. In contrast, randomized TTO studies were heterogeneous in product concentration, dosing regimen, and sham comparators and did not demonstrate a statistically significant effect on mite density or mite eradication versus sham comparators.

## 1 Introduction

*Demodex* blepharitis is an inflammatory disease of the eyelid margins caused by infestation with *Demodex* mites, specifically, *Demodex folliculorum* and *Demodex brevis* (1). These ectoparasites inhabit eyelash follicles and sebaceous glands, where they contribute to lid margin and ocular surface disease by inducing chronic and progressive clinical manifestations including misdirected lashes and madarosis, erythema, meibomian gland disease, worse dry eye disease, blepharokeratitis and corneal scarring (2). Patients often complain of eyelid itching, fluctuating vision, epiphora, redness, and irritation (3). The presence of collarettes is a pathognomonic sign of *Demodex* blepharitis, with several robust studies demonstrating that eyes exhibiting collarettes are consistently positive for *Demodex* mites (4–6). Typically identified on slit-lamp examination, a higher number of collarettes has been associated with more severe mite infestation (4).

*Demodex* blepharitis has increasingly been recognized as a prevalent and underdiagnosed contributor to chronic eyelid irritation and increased patient burden (6, 7). More than half (58%) of US patients presenting to eye clinics for various reasons exhibit symptoms of *Demodex* blepharitis (6). In Europe, the evidence base is more limited, but a recent study conducted by Nanavaty et al. (2026) (8) showed a similar prevalence rate of 54%. Patients frequently report a substantial negative impact on daily functioning, with limitations in reading, driving, household tasks, workplace productivity, and tolerance of glasses or contact lenses (7, 9).

In 2023, XDEMVY^®^ (lotilaner ophthalmic solution, 0.25% [lotilaner 0.25%]) became the first and only FDA-approved therapy for *Demodex* blepharitis. Evidence from replicate, randomized, double-blind controlled trials demonstrated that patients treated with lotilaner 0.25% experienced significantly higher rates of clinical improvement across multiple endpoints, including collarette reduction to 0–2 collarettes, mite eradication, and erythema cure (10, 11). Off-label therapeutic approaches for *Demodex* blepharitis remain heterogeneous and largely empirical. Conventional management strategies typically focus on eyelid hygiene, including lid scrubbing to mechanically remove collarettes and accumulated mite debris from the eyelid margins (12). Other off-label topical agents such as tea tree oil and its derivatives, including terpinen-4-ol (collectively referred to as TTO products in this study) have been employed at varying concentrations for their purported acaricidal activity; however, evidence supporting their efficacy is limited and inconsistent, and some forms of TTO have even been shown to be harmful to meibomian gland epithelial cells under continuous exposure to the active component across a concentration range of 1.0%–0.001% *in vitro* (13, 14). Additional methods include in-office mechanical exfoliation and device-based procedures, while more refractory cases have prompted the use of adjunctive procedures such as microblepharoexfoliation (MBE) or intense pulsed light (IPL) (15). However, clinical evidence evaluating the use of these therapies in clinical practice is sparse.

The objective of this systematic literature review (SLR) and meta-analysis was to evaluate the quality of evidence and comparative treatment effects of lotilaner 0.25% versus vehicle and TTO interventions versus sham in patients with *Demodex* blepharitis.

## 2 Materials and methods

### 2.1 Data sources and search strategy

This SLR and meta-analysis was conducted in accordance with the Preferred Reporting Items for Systematic Reviews and Meta-Analyses (PRISMA) guidance for evidence synthesis and decision-making (16). MEDLINE (via PubMed), EMBASE, Cochrane CENTRAL, ClinicalTrials.gov, and the International Clinical Trials Registry Platform (ICTRP) were searched from database inception to July 10, 2025, to identify studies evaluating lotilaner 0.25% and TTO interventions (tea tree oil or terpinen-4-ol) for the treatment of *Demodex* blepharitis. In addition, conference abstracts published in journal supplements and retrievable via PubMed or EMBASE from 2023 to 2025 were also included.

### 2.2 Study selection and data extraction

Trials were deemed eligible for inclusion if they evaluated the treatment effects of lotilaner 0.25% or TTO interventions and included adult patients (≥18 years) with *Demodex* infestation of the eyelashes or eyelids, with or without a formal diagnosis of *Demodex* blepharitis. Studies with mixed populations were included if results were reported stratified by *Demodex* infestation status. In such cases, only the relevant subset of patients was retained for inclusion in the SLR and meta-analysis.

Only randomized controlled trials (RCTs) published in English were included. Studies were excluded if they reported qualitative findings without quantitative data or lacked sufficient information to extract relevant outcomes. We also excluded studies that examined patients with blepharitis in absence of *Demodex* mites in eyelashes. For the purpose of this study, tea tree oil and terpinen-4-ol were treated as sufficiently similar to allow pooling of results across outcomes.

Records identified in the search were dual-screened, using a two-stage process comprising title and abstract screening, followed by full-text review. Data extraction was performed by two independent investigators. Discrepancies or conflicts were resolved in consultation with a third independent investigator, as needed.

## 3 Study outcomes

The outcomes of interest included mite density, mite eradication, collarette reduction to 0–2 collarettes (Grade 0), and erythema cure. Results were pooled for 2–4 weeks and 6–8 weeks. If trial results from an RCT were available at both 2 and 4 weeks, or 6 and 8 weeks, the longer period was selected for inclusion in the analysis.

## 4 Quality assessment

The study quality of the included RCTs was assessed using the Risk of Bias Tool (RoB2) (17).

## 5 Statistical analysis

Study and patient characteristics were summarized descriptively. Meta-analyses of treatment effects on the outcomes of mite density, mite eradication, collarette reduction to 0–2 collarettes (Grade 0), and erythema cure were conducted using data from RCTs comparing active interventions for *Demodex* blepharitis with placebo, vehicle, or sham controls.

Pooled mean differences and odds ratios with corresponding 95% confidence intervals were calculated, and the results were displayed in forest plots. Heterogeneity was assessed using Higgins’ I². In the presence of substantial heterogeneity arising from underlying differences in study populations, we used random effects models as the base case. Meta-analyses were conducted using R statistical software.

## 6 Results

Of 1,197 records initially identified through database searches, 845 underwent title/abstract screening after de-duplication, and 173 were retained for full text screening, of which 164 were reviewed for relevance. Full texts of nine studies were not retrieved because no full text articles were available and they were not eligible for inclusion based on the title/abstract alone. Nine unique RCTs comprising 1,513 patients were identified for inclusion in the SLR and meta-analysis. Of those, four evaluated lotilaner 0.25% (10, 11, 18, 19) and five evaluated TTO (20–24) (**Figure 1**).

**Figure 1:**
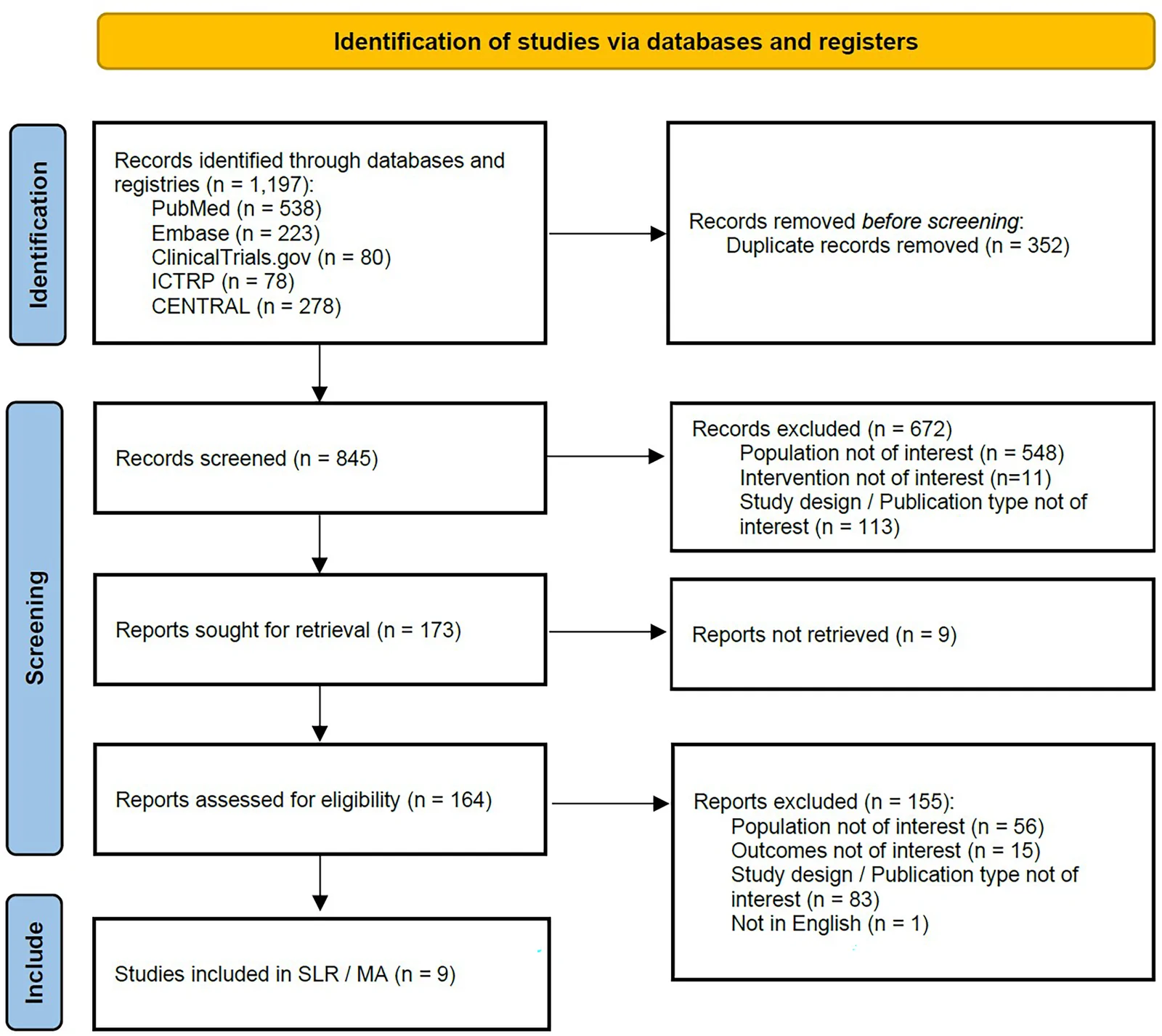
PRISMA diagram.

The included studies were conducted across a variety of countries/regions including the US (n=3), Türkiye (n=3), Mexico (n=2), and South Korea (n=1). Study and patient characteristics are presented in **Table 1**. Across the four lotilaner 0.25% trials, mean age ranged from approximately 59 to 68 years, and the proportion of male participants ranged from 23% to 58%. The TTO trials enrolled a wider age range across studies, with mean ages spanning approximately 28 to 76 years; two Türkiye-based trials (20, 24) enrolled notably younger cohorts (mean age ~28–34 years) compared with the other included studies. The proportion of male participants in the included TTO trials ranged from 34% to 71%.

**Table 1:** Study and Patient Characteristics.

Reference (Study)	Intervention	N	Product concentration [dosing frequency]	DoT	Country	Age, mean years (SD)	Male, %	Mite density^a^ at baseline, mean (SD)	Collarette grade, mean (SD)	
									At baseline	Outcome definition
Lotilaner 0.25%										
Gaddie 2023 (Saturn-2) (10)	lotilaner 0.25%	203	TP-03, 0.25% [BID]	43 days	US	63.9 (15.15)	52.2	3.16 (1.42)	2.9 (0.77)	Grade 0 to 4^c^
	vehicle	209	Vehicle [BID]			65.1 (13.35)	50.7	3.33 (1.71)	3.0 (0.80)	
Gonzalez-Salinas 2022 (Jupiter) (18)	lotilaner 0.25%	30	TP-03, 0.25% [BID]	28 days	Mexico	59.6 (2.1)	23.3	2.7 (0.16)^b^	3.6 (0.14)^b^	Grade 0 to 4^c^
	vehicle	30	Vehicle [BID]			61.7 (1.9)	40.0	2.9 (0.16)^b^	3.6 (0.11)^b^	
Yeu 2023a (Saturn-1) (11)	lotilaner 0.25%	212	TP-03, 0.25% [BID]	43 days	US	66.1 (12.1)	42.0	3.19 (1.67)	2.8 (0.77)	Grade 0 to 4^c^
	vehicle	209	Vehicle [BID]			67.8 (12.6)	44.0	3.16 (1.59)	2.8 (0.71)	
Yeu 2023b (Europa) (19)	lotilaner 0.25%	27	TP-03, 0.25% [BID]	42 days	Mexico	58.5 (SEM: 14.1)	36	3.27 (SEM: 0.35)	3.16 (SEM: 0.19)	Grade 0 to 4^c^
	vehicle	27	Vehicle [BID]			62.1 (SEM: 8.3)	58.3	2.95 (SEM: 0.25)	3.33 (0.16)	
TTO										
Arici 2022 (NCT04441528) (20)	T4O + sodium hyaluronate lid wipes	24	NR [BID]	8 weeks	Türkiye	29.2 (11.8)	50.0	1.52 (1.54)	NR	NR
	baby shampoo	24	NR [BID]			33.7 (12.5)	41.7	1.21 (0.95)	NR	
Epstein 2020 (21) ^d^	T4O scrub + MBE^e^	26	NR [BID]	2 months	US	71.0 (5.8)	46.2	0.9 (0.75)^f,g^	NR	NR
	sham scrub + MBE^e^	24	Sham [BID]			75.6 (5.0)	70.8	0.75 (0.5)^f,g^	NR	
Karakurt 2018 (22)	TTO eyelash shampoo	75	TTO 7.5% [BID]	4 weeks	Türkiye	57.52 (14.22)	46.7	10.25 (NR)	2.03 (NR)	NR
	conventional eyelash shampoo	60	Sham [BID]			55.15 (13.97)	41.7	10.82 (NR)	1.83 (NR)	
Koo 2012 (23)	TTO eyelid scrub	141	TTO 10% + 50% [QD/QW]	1 month	South Korea	53.7 (10.3)	34.0	0.4 (0.81)^f,h^	NR	NR
	sham eyelid scrub	140	Sham [QD/QW]			55.6 (11.3)	37.0	0.525 (0.884)^f,h^	NR	
Mergen 2021 (NCT04678531) (24)	TTO + chamomile oil swabs	26	TTO 7.5% [BID]	8 weeks	Türkiye	28.4 (5.2)	46.2	1.45 (1.14)	NR	NR
	baby shampoo	26	NR [BID]			31.8 (11.0)	43.5	1.20 (0.97)	NR	

BID, twice daily; DoT, duration of treatment; MBE, microblepharoexfoliation; N, number of patients; NCT, ClinicalTrials.gov identifier; NR, not reported; QD, once daily; QW, once weekly; SD, standard deviation; SEM, standard error of the mean; T4O, terpinen-4-ol; TP-03, lotilaner ophthalmic solution, 0.25%; TTO, tea tree oil; US, United States.

^a^ Defined as number of mites per lash.

^b^ Digitized.

^c^ 0 = 0–2 lashes/eyelid, 1 = 3–10 lashes/eyelid, 2 = >10 or <1/3 lashes/eyelid, 3 = ≥1/3 to <2/3 lashes/eyelid, 4 = ≥2/3 lashes/eyelid.

^d^ Blinded in the first month, open-label in the second month.

^e^ MBE performed at baseline and 1-month follow-up.

^f^ To enable inclusion in the meta-analyses, mite density was standardized to represent the number of mites per lash.

^g^ Data was originally presented as number of mites in four lashes.

^h^ Data was originally presented as number of mites in eight lashes.

### 6.1 Drug formulation and concentration

Lotilaner trials (n=4) used the same drug formulation and dosing regimen, with lotilaner 0.25% administered twice daily (BID) to the treatment eye(s). Among TTO trials, substantial variability in dosing regimens was observed, driven by differences in formulation, concentration, and modes of application. TTO concentrations ranged from 7.5–50%, although this was infrequently reported (22–24). Moreover, studies often combined TTO with another treatment modality or active ingredient (20, 21, 24). The most common dosing frequency was BID, although some studies used mixed schedules of once daily (QD)/once weekly (QW) dosing.

### 6.2 Outcome scales and definitions

Among studies included in the meta-analysis, mite eradication was consistently defined as the complete absence of mites, corresponding to 0 mites per lash. The most common metric for assessing mite density was mites per lash, representing the average number of *Demodex* mites identified per eyelash examined. Two trials reported mite counts per 4–8 lashes (21, 23). Collarette severity was consistently graded on a scale from 0 (≤2 affected lashes per eyelid or no visible collarettes) to 4 (≥two-thirds of the lash line is affected), based on the number of lashes affected or the proportion of the lashes involved. Collarette severity grade 0 was defined as ≤2 affected lashes per eyelid (10, 11, 19) or the complete absence of collarettes (18). Erythema severity was assessed on a scale from 0 (normal age-related lid coloration) to 3 (deep red, diffuse confluent capillary redness present along the lid edge), with erythema cure defined as achieving an erythema grade of 0 on the upper eyelid of the analysis eye (10, 11).

### 6.3 Study quality

Quality assessment was performed on all studies included in the meta-analysis, of which five trials were rated as good quality and four were considered to have at least some concerns regarding risk of bias (**Figure 2**). The lotilaner 0.25% trials were generally robust: three trials were judged to be at low risk of bias and one at some risk. In contrast, the overall study quality of RCTs assessing TTO interventions was lower. Of the five trials assessing TTO, two were rated as good quality, one had some risk of bias, and two were assessed as having high risk of bias.

**Figure 2:**
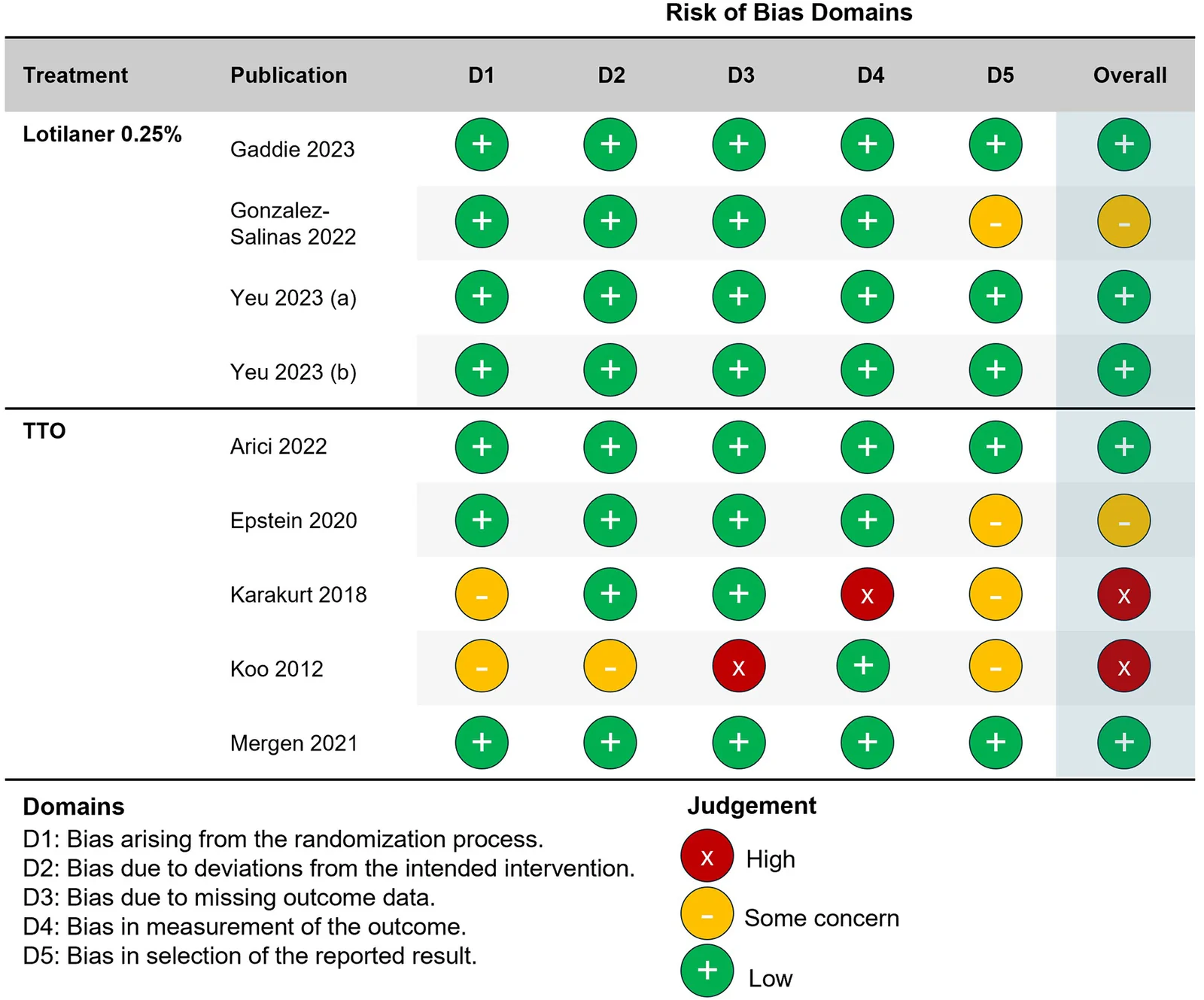
Risk of bias.

### 6.4 Clinical outcomes

#### 6.4.1 Mite density (mites/lash)

All four RCTs evaluating lotilaner 0.25% reported mite density outcomes, as did four of the five trials evaluating TTO. Two TTO studies (Epstein 2020, Koo 2012) reported mite density as the total number of mites across all lashes assessed (21, 23). To enable inclusion in the meta-analyses, mite density was standardized across studies to represent the number of mites per lash.

Patients treated with lotilaner 0.25% had significantly lower mite density than those receiving vehicle at both timepoints assessed (p<0.0001). The mean difference was -1.29 at 2–4 weeks (95% CI: -1.44, -1.15), and this was maintained at 6–8 weeks (95% CI: -1.47, -1.12) (**Figures 3**, **4**). Conversely, TTO did not show a meaningful difference from sham in mite density at either timepoint. At 2–4 weeks the mean difference was -0.02 (95% CI: -0.15, 0.11; p=0.747), which reduced to no difference at 6–8 weeks (mean difference: -0.00 [95% CI: -0.03, 0.02]; I^2^: 0.0%; p=0.651) (**Figures 3**, **4**).

**Figure 3:**
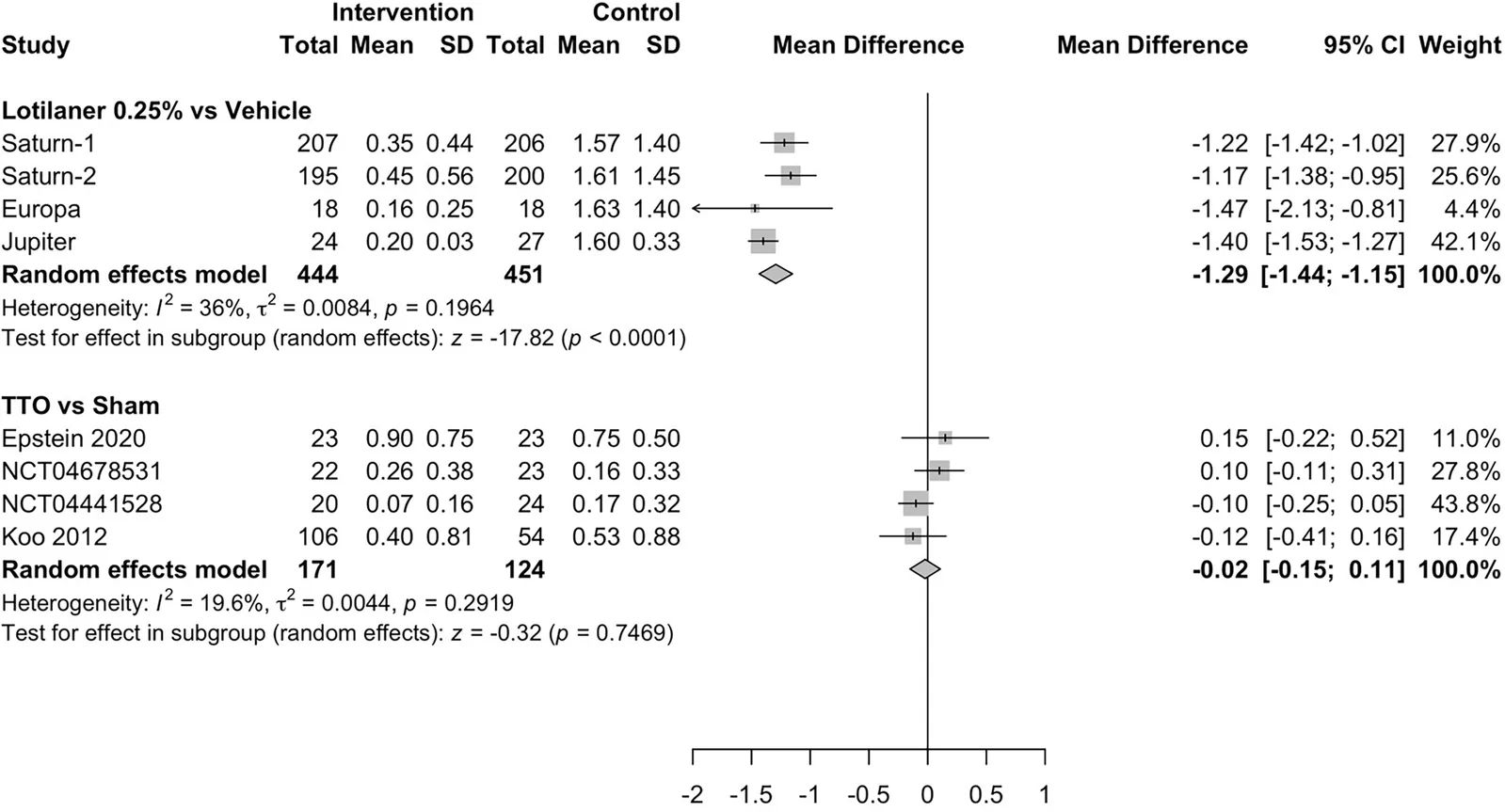
Mite density, 2–4 weeks. CI, confidence interval; TTO, tea tree oil; SD, standard deviation; vs, versus.

**Figure 4:**
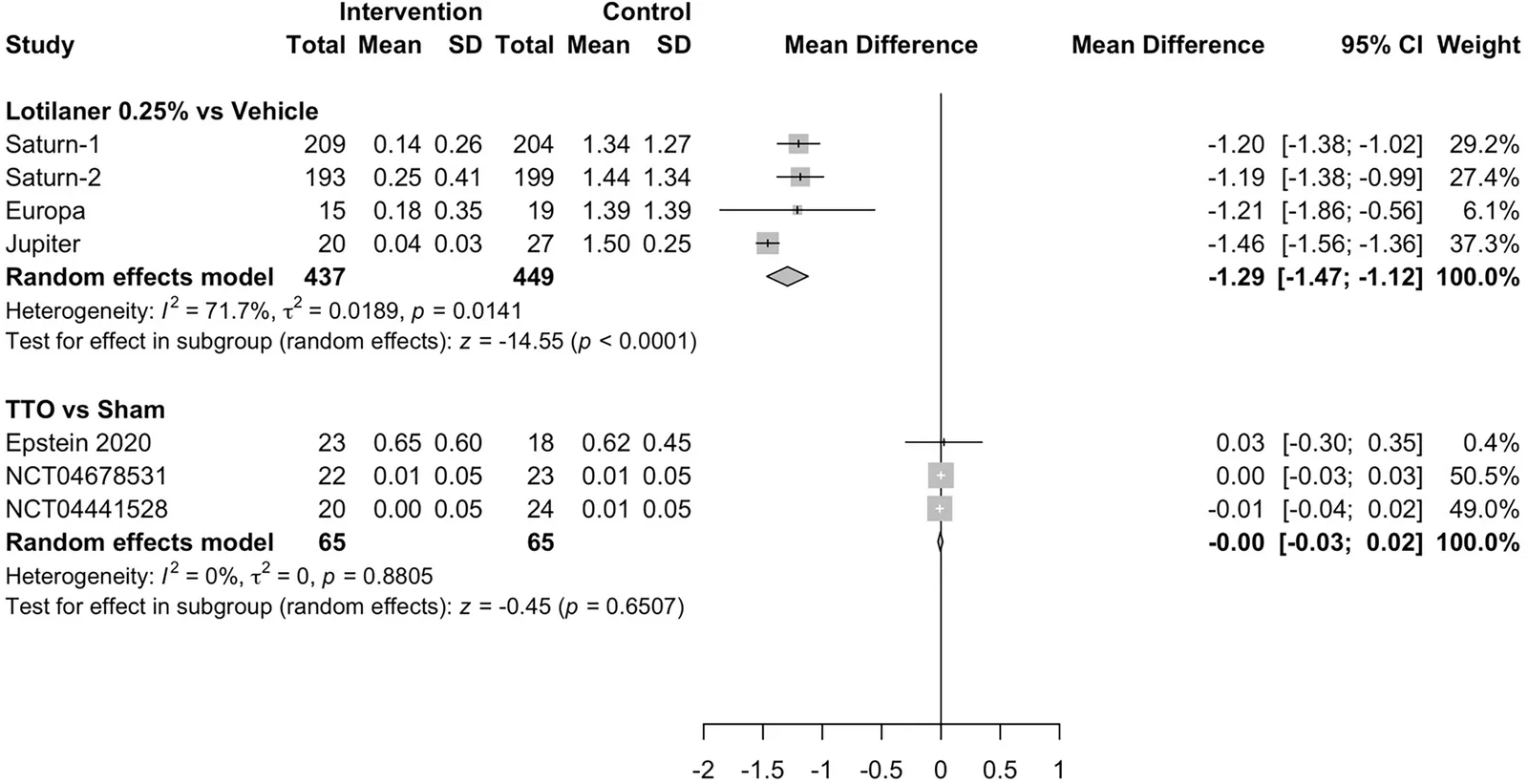
Mite density, 6–8 weeks. CI, confidence interval; TTO, tea tree oil; SD, standard deviation; vs, versus.

#### 6.4.2 Mite eradication

Mite eradication, defined as the complete absence of mites (0 mites/lash) was reported at both timepoints in all four lotilaner 0.25% trials and in four of the five TTO trials at 2–4 weeks; two TTO trials reported mite eradication at 6–8 weeks.

Patients treated with lotilaner 0.25% were significantly more likely to achieve mite eradication than those receiving vehicle at both timepoints assessed (p<0.0001), with ORs of 4.70 (95% CI: 3.35, 6.59) at 2–4 weeks and 9.60 (95% CI: 5.50, 16.74) at 6–8 weeks (**Figures 5**, **6**). TTO did not differ meaningfully from sham in mite eradication at either timepoint. At 2–4 weeks the OR was 1.81 (95% CI: 0.62, 5.26; p=0.274), declining to 1.47 (95% CI: 0.17, 12.51; p=0.722) at 6–8 weeks (**Figures 5**, **6**).

**Figure 5:**
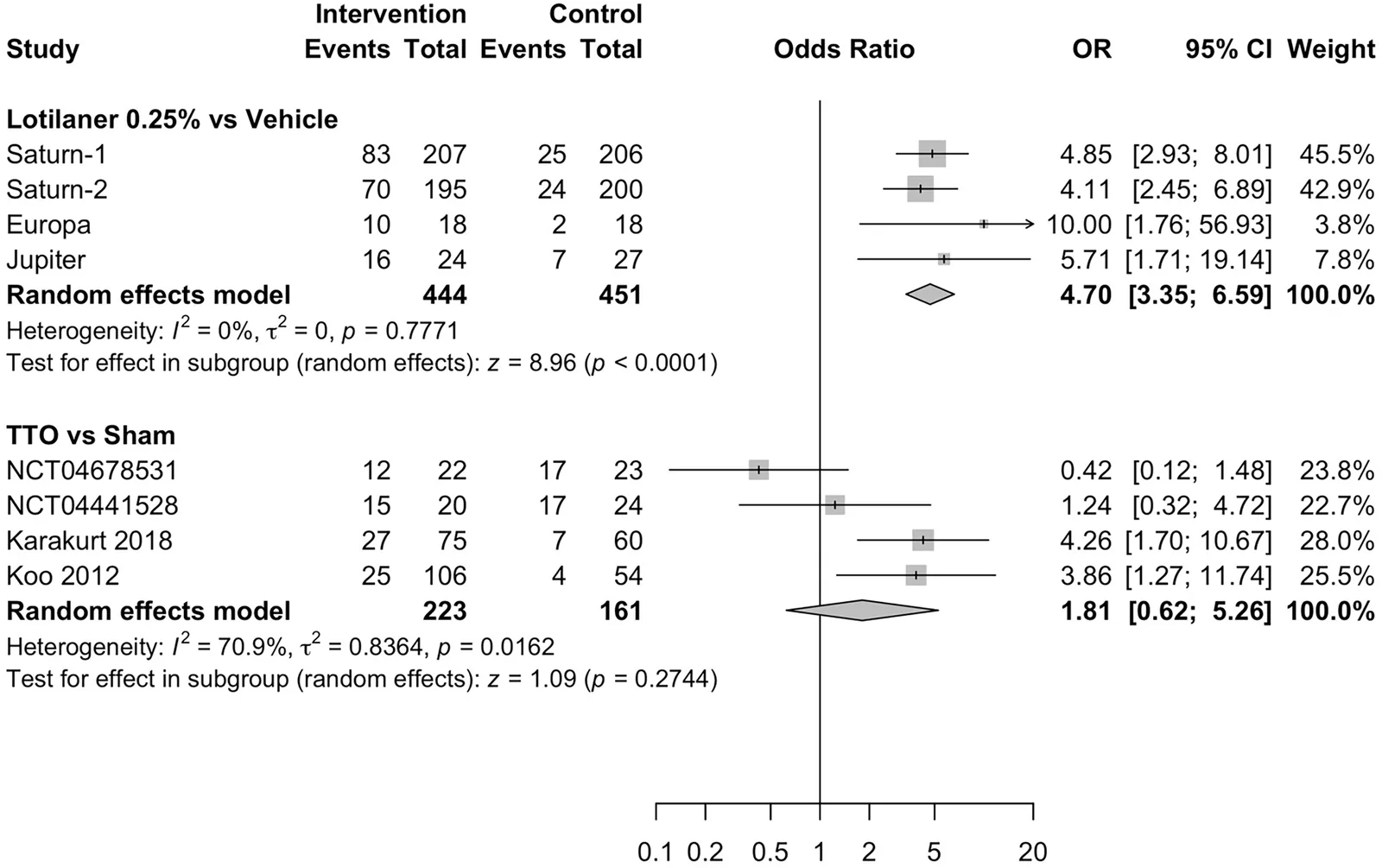
Mite eradication, 2–4 weeks. CI, confidence interval; OR, odds ratio; TTO, tea tree oil; vs, versus.

**Figure 6:**
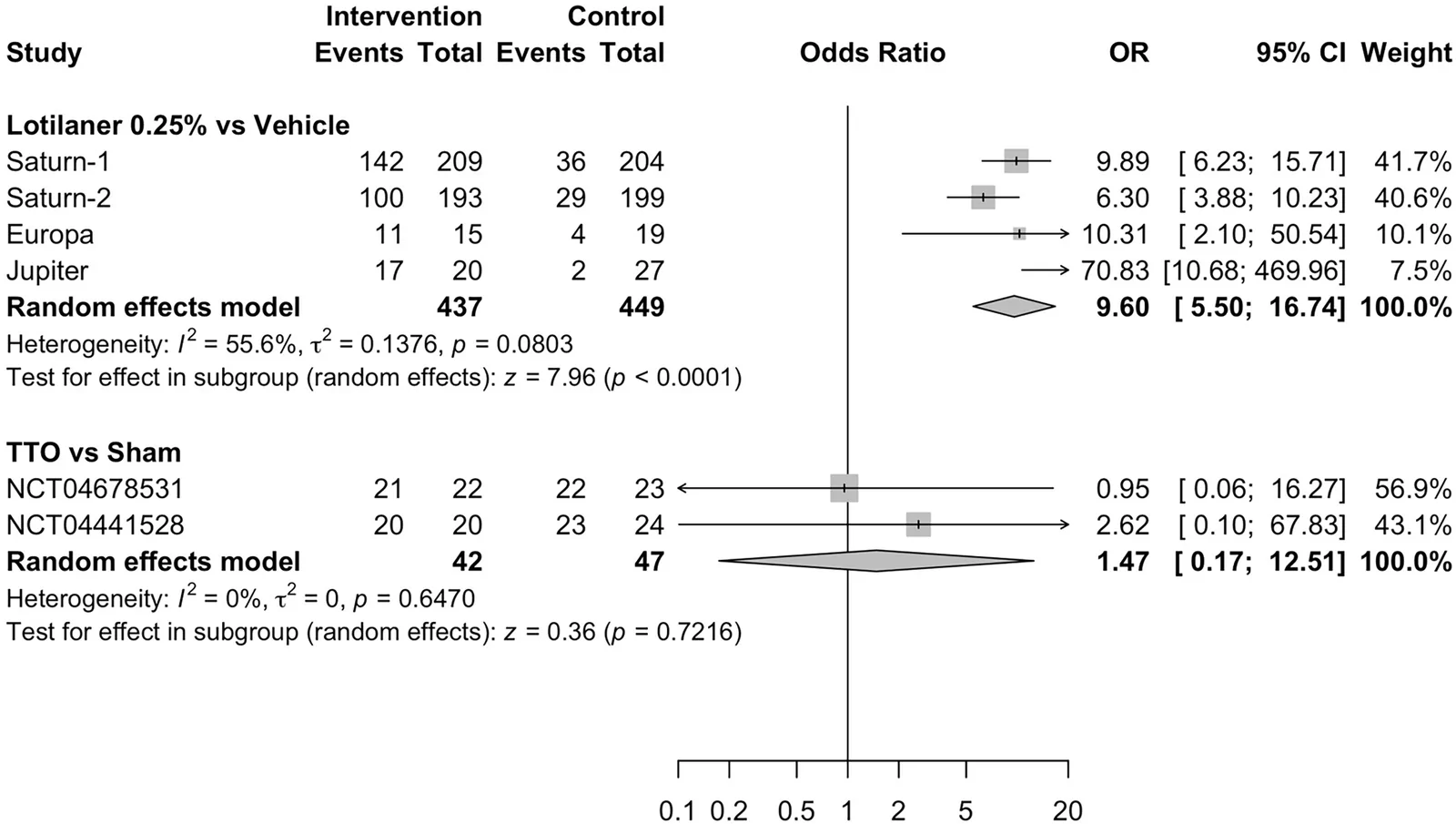
Mite eradication, 6–8 weeks. CI, confidence interval; OR, odds ratio; TTO, tea tree oil; vs, versus.

#### 6.4.3 Collarette reduction to 0–2 collarettes

Collarette reduction to 0–2 collarettes was reported in three RCTs comparing lotilaner 0.25% with vehicle. None of the TTO trials reported collarette reduction at either timepoint.

Patients treated with lotilaner 0.25% showed significantly higher rates of collarette reduction to 0–2 collarettes than those treated with vehicle, with rates further improving over time (**Figure 7**). At 2–4 weeks, patients treated with lotilaner 0.25% had 8.39 times the odds of achieving collarette reduction to 0–2 collarettes than those treated with vehicle (95% CI: 4.57, 15.40; p<0.0001). The odds further increased to 9.67 (95% CI: 6.64, 14.08; p<0.0001) at 6–8 weeks of follow-up.

**Figure 7:**
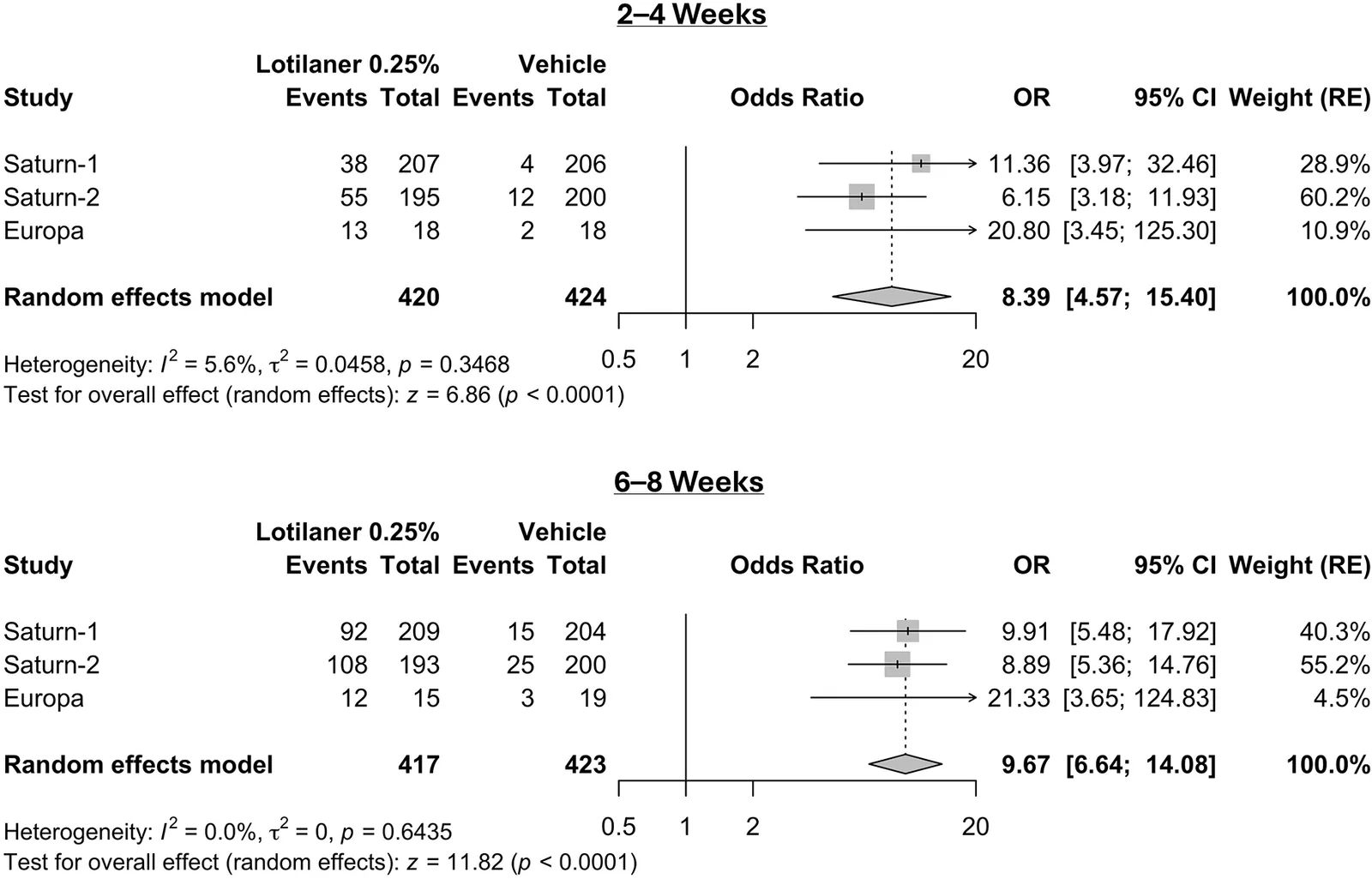
Collarette reduction to 0–2 collarettes. CI, confidence interval; OR, odds ratio.

#### 6.4.4 Erythema cure (grade 0 erythema)

Erythema cure was reported in two RCTs comparing lotilaner 0.25% with vehicle. Overall, patients treated with lotilaner 0.25% had significantly higher odds of erythema cure than those treated with vehicle at 6–8 weeks of follow-up (OR 3.91 [95% CI: 2.55, 5.99; I^2^ = 0.0%; p<0.0001]) (**Figure 8**).

**Figure 8:**
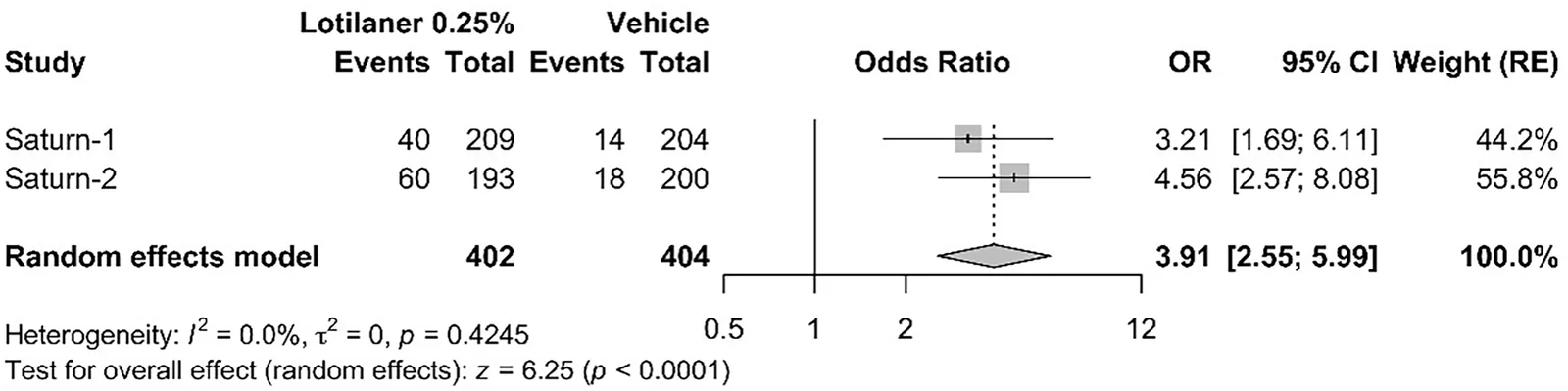
Erythema cure, 6–8 weeks. CI, confidence interval; OR, odds ratio.

## 7 Discussion

This meta-analysis synthesized the available evidence from vehicle- or sham-controlled RCTs to generate summary estimates across a range of relevant clinical outcomes. To the best of our knowledge, no recently published studies have conducted a similarly rigorous evaluation of the available clinical evidence from vehicle- or sham-controlled trials of lotilaner 0.25% and commonly used management options containing TTO.

Lotilaner 0.25% demonstrated consistent and statistically significant improvements in mite density, with significantly higher rates of mite eradication and collarette reduction to 0–2 collarettes at both 2–4 weeks and 6–8 weeks, as well as erythema cure at 6–8 weeks of follow-up, compared with vehicle (p<0.0001). In contrast, TTO did not demonstrate a significant benefit over sham across any of the evaluated outcomes. A formal indirect treatment comparison or network meta-analysis was not undertaken and, as such, these findings should not be interpreted as demonstrating direct superiority of lotilaner 0.25% over TTO.

Patients treated with lotilaner 0.25% experienced a marked reduction in mite density, with a mean difference of -1.29 mites/lash versus vehicle at both 2–4 weeks (95% CI: -1.44, -1.15) and 6–8 weeks (95% CI: -1.47, -1.12; p<0.0001 at both timepoints). This stability across timepoints is consistent with the rapid onset of acaricidal activity observed in the pivotal trials (Saturn-1, Saturn-2), where statistically significant reductions in mite density were apparent as early as two weeks (10, 11). The magnitude of the effect on mite eradication increased over time. Patients treated with lotilaner 0.25% had 4.70 times the odds of achieving complete mite eradication (0 mites/lash) at 2–4 weeks (95% CI: 3.35, 6.59) and 9.60 times the odds at 6–8 weeks (95% CI: 5.50, 16.74), reflecting the cumulative effect of sustained twice-daily dosing on the mite life cycle.

A similar pattern was observed for collarette reduction to 0–2 collarettes, with an odds ratio of 8.39 (95% CI: 4.57, 15.40) at 2–4 weeks and 9.67 (95% CI: 6.64, 14.08) at 6–8 weeks. Erythema cure was achieved at nearly fourfold higher odds with lotilaner 0.25% than with vehicle at 6–8 weeks (OR 3.91; 95% CI: 2.55, 5.99). These results are consistent with the pooled analysis of Saturn-1 and Saturn-2 reported by Yeu et al. (2025), which demonstrated comparable improvements across mite density, mite eradication, collarette reduction, and erythema cure, supporting the reproducibility of the treatment effect of lotilaner 0.25% (25). Between-study heterogeneity across lotilaner 0.25% trials was low (I² = 0%) for erythema cure and modest for the remaining endpoints, indicating concordant treatment effects across Jupiter, Europa, Saturn-1, and Saturn-2 despite differences in trial size, geography, and follow-up duration (10, 11, 18, 19). This concordance is notable given that the four trials enrolled patients across two countries, used center-specific examiners, and predated harmonization of FDA endpoint definitions.

In contrast, TTO had no significant effect on mite density relative to sham, with mean differences of -0.02 mites/lash (95% CI: -0.15, 0.11; p=0.747) at 2–4 weeks and no difference in mites/lash (95% CI: -0.03, 0.02; p=0.651) at 6–8 weeks. A similar pattern was observed for mite eradication, where the odds of achieving complete eradication did not differ significantly between TTO and sham at either timepoint (OR 1.81 [95% CI: 0.62, 5.26; p=0.274] at 2–4 weeks; OR 1.47 [95% CI: 0.17, 12.51; p=0.722] at 6–8 weeks). The wide confidence intervals reflect the small sample sizes and methodological heterogeneity of the underlying TTO trials. Taken together, the findings from this SLR and meta-analysis reinforce the importance of using well-defined, quantifiable endpoints, such as mite density, mite eradication, and collarette reduction to 0–2 collarettes to assess true therapeutic effects in *Demodex* blepharitis, as well as erythema cure as an indicator for the reduction or resolution of eyelid inflammation.

The findings for lotilaner 0.25% were supported by evidence derived from multiple, well-designed RCTs that utilized standardized diagnostic criteria, consistent treatment regimens, and clinically relevant outcome measures. In contrast, the TTO evidence base was constrained by considerable variability in drug formulations and concentrations, adjunctive interventions, and substantial heterogeneity in comparators, as well as small sample sizes and heterogeneous study populations across studies. The considerable heterogeneity observed in the TTO pooled estimates is consistent with the substantial variability in formulations, comparators, and adjunctive interventions across the five included TTO trials.

For instance, in Koo 2012, a dual-concentration protocol was employed, combining daily lid scrubs with 10% TTO with weekly in-clinic lid scrubs with 50% TTO (23). Whereas all four lotilaner RCTs used a true vehicle control, the TTO trials compared TTO against baby shampoo (20, 24), TTO-free eyelash shampoo (22), or sham scrubs administered alongside in-office MBE in both arms (21). Furthermore, in addition to the generally small sample sizes (<30 patients per arm in most trials), TTO trials enrolled patient populations with heterogeneous baseline severity, ranging from ~0.5 (23) to ~10 mites/lash (22) compared to lotilaner 0.25% trials, where baseline mite density was generally ~3 mites/lash (10, 11, 18, 26). These differences complicate the comparability of clinical endpoints across TTO studies, as patients beginning treatment with a lower relative mite burden require fewer mites to be cleared to reach mite eradication. This was particularly evident in two studies with low baseline mite density (20, 24), where almost all patients achieved mite eradication at 6–8 weeks of follow-up in both the intervention and comparator groups.

These findings indicate that, based on the available randomized evidence, TTO does not produce a statistically meaningful reduction in *Demodex* mite burden compared with ‘sham’. Given the methodological heterogeneity of TTO studies, the pooled effect for TTO versus ‘sham’ should be interpreted as a failure to outperform active lid hygiene regimens in heterogeneous populations, rather than as definitive evidence regarding the effects of TTO on *Demodex* mites, which remain uncertain.

Confidence in the pooled TTO estimates was further undermined by study quality: the majority of the TTO trials were rated as having at least some concerns or a high risk of bias under RoB2, most often driven by issues in the randomization process, deviations from intended interventions, and selective reporting of outcomes (21–23). The lotilaner 0.25% trials on the other hand were predominantly rated as low risk of bias, lending substantially greater confidence to the magnitude and direction of the observed effects (10, 11, 18, 19). Furthermore, while dosing regimens for lotilaner 0.25% were consistent across trials, they were highly heterogeneous in the TTO trials, with substantial variability in formulations, concentrations, routes of administration, and dosing frequencies (QD–BID).

Our findings for lotilaner 0.25% are directionally consistent with two prior meta-analyses limited to lotilaner 0.25% conducted by Abo Zeid (2024) (27) and Talha (2024) (28), both of which reported reduction in mite density and substantially elevated relative risks of mite eradication and meaningful collarette reduction versus vehicle. Another review pooled different off-label and over-the-counter options for *Demodex* blepharitis, including TTO-based treatments (Navel et al. [2019]) (1). Our analysis advances this prior work by: 1) evaluating lotilaner 0.25% and TTO in parallel; 2) including all four vehicle-controlled lotilaner 0.25% RCTs (Jupiter, Europa, Saturn-1, Saturn-2) with an extended search timeframe (through July 2025); and 3) stratifying outcomes by follow-up intervals (2–4 and 6–8 weeks), enabling evaluation of both the onset and persistence of treatment effects rather than a single pooled estimate. Given that *Demodex* blepharitis is a disease that will not remit or resolve on its own, therapies with consistent and durable mite-killing activity are likely to provide disease control, and thus better long-term symptom relief (25, 29). The consistency and magnitude of the observed treatment effects support the therapeutic use of lotilaner 0.25% as a first-line pharmacologic therapy for *Demodex* blepharitis. The evidence from randomized TTO studies was limited and considerably heterogeneous and did not demonstrate a statistically significant effect of TTO versus sham in mite density reduction or mite eradication. Together, these findings provide important context around the extent and quality of evidence available for both lotilaner 0.25% and TTO in this patient population, particularly given the lack of direct comparative data.

## 8 Limitations

The results of this meta-analysis should be interpreted in light of a few limitations. The meta-analysis is informed by an SLR with a search cutoff of July 10, 2025; therefore, any evidence published after this date was not included. Analyses for both lotilaner 0.25% and TTO were feasible only for mite eradication and mite density, as these were the sole outcomes consistently reported across studies. No direct treatment comparison or network meta-analysis of lotilaner 0.25% versus TTO was performed; therefore, the findings reflect separate comparisons with their respective comparators. Other clinically relevant endpoints, such as collarette reduction to 0–2 collarettes and erythema cure, were inconsistently documented for TTO, limiting the ability to conduct a meta-analysis for TTO for these outcomes. Additionally, pooled TTO estimates should be interpreted in the context of considerable heterogeneity in within-trial concentrations (7.5–50%) and comparators (baby shampoo, TTO-free shampoo, sham scrubs with concurrent MBE). The wide range of concentrations and active comparators employed in TTO studies may represent a confounding factor and should be considered when interpreting the efficacy of TTO in these studies.

Furthermore, variability in diagnostic criteria or sampling methods (e.g., lash manipulation techniques) may have introduced heterogeneity in reported mite densities or eradication outcomes. The geographic concentration of trials may limit the generalizability of findings to populations with different environmental exposures, ethnic backgrounds, or ocular surface characteristics. Lastly, publication bias cannot be ruled out, particularly among non-registered trials evaluating off-label TTO-based treatments.

## 9 Conclusions

Lotilaner 0.25% showed consistent and statistically significant improvements across all efficacy outcomes assessed (mite density reduction, mite eradication, collarette reduction to 0–2 collarettes, and erythema cure), supported by high-quality clinical evidence demonstrating the therapeutic role of lotilaner 0.25% as an efficacious pharmacologic treatment for *Demodex* blepharitis. Meanwhile, the evidence base for TTO, which was limited by substantial methodological variability, did not show a statistically significant difference versus sham for mite density reduction or mite eradication. Given the burden of *Demodex* blepharitis and its high prevalence in clinical practice, the availability of an FDA-approved, consistently effective therapy represents a significant step forward in patient care.

## Data Availability

The original contributions presented in the study are included in the article, further inquiries can be directed to the corresponding author/s.
